# Common and distinct patterns of intrinsic brain activity alterations in major depression and bipolar disorder: voxel-based meta-analysis

**DOI:** 10.1038/s41398-020-01036-5

**Published:** 2020-10-19

**Authors:** Jiaying Gong, Junjing Wang, Shaojuan Qiu, Pan Chen, Zhenye Luo, Jurong Wang, Li Huang, Ying Wang

**Affiliations:** 1 Medical Imaging Center, First Affiliated Hospital of Jinan University, Guangzhou, 510630 China; 2 Department of Radiology, Six Affiliated Hospital of Sun Yat-sen University, Guangzhou, 510655 China; 3 Department of Applied Psychology, Guangdong University of Foreign Studies, Guangzhou, 510006 China

**Keywords:** Epigenetics and behaviour, Bipolar disorder

## Abstract

Identification of intrinsic brain activity differences and similarities between major depression (MDD) and bipolar disorder (BD) is necessary. However, results have not yet yielded consistent conclusions. A meta-analysis of whole-brain resting-state functional MRI (rs-fMRI) studies that explored differences in the amplitude of low-frequency fluctuation (ALFF) between patients (including MDD and BD) and healthy controls (HCs) was conducted using seed-based *d* mapping software. Systematic literature search identified 50 studies comparing 1399 MDD patients and 1332 HCs, and 15 studies comparing 494 BD patients and 593 HCs. MDD patients displayed increased ALFF in the right superior frontal gyrus (SFG) (including the medial orbitofrontal cortex, medial prefrontal cortex [mPFC], anterior cingulate cortex [ACC]), bilateral insula extending into the striatum and left supramarginal gyrus and decreased ALFF in the bilateral cerebellum, bilateral precuneus, and left occipital cortex compared with HCs. BD showed increased ALFF in the bilateral inferior frontal gyrus, bilateral insula extending into the striatum, right SFG, and right superior temporal gyrus (STG) and decreased ALFF in the bilateral precuneus, left cerebellum (extending to the occipital cortex), left ACC, and left STG. In addition, MDD displayed increased ALFF in the left lingual gyrus, left ACC, bilateral precuneus/posterior cingulate gyrus, and left STG and decreased ALFF in the right insula, right mPFC, right fusiform gyrus, and bilateral striatum relative to BD patients. Conjunction analysis showed increased ALFF in the bilateral insula, mPFC, and decreased ALFF in the left cerebellum in both disorders. Our comprehensive meta-analysis suggests that MDD and BD show a common pattern of aberrant regional intrinsic brain activity which predominantly includes the insula, mPFC, and cerebellum, while the limbic system and occipital cortex may be associated with spatially distinct patterns of brain function, which provide useful insights for understanding the underlying pathophysiology of brain dysfunction in affective disorders, and developing more targeted and efficacious treatment and intervention strategies.

## Background

Affective disorders, such as major depressive disorder (MDD, or unipolar depression) and bipolar disorder (BD), are highly prevalent and debilitating conditions associated with high suicide rates and a heavy social burden^1^. BD is characterized by recurrent depressive and manic/hypomanic episodes, and the depressive episodes are the most common mood manifestation of the illness^2^. Approximately 69% of patients with BD are initially misdiagnosed with MDD, and patients meet with an average of four physicians before they are correctly diagnosed with BD^3^, leading to inappropriate treatment, poor clinical outcomes and greater healthcare costs. The identification of objective biomarkers based on neuroimaging techniques will not only facilitate more accurate differential diagnosis between the two affective disorders, but will also advance our understanding of the pathophysiological mechanisms underlying MDD and BD.

Neuroimaging evidence has identified brain structural and functional alterations in multiple neuronal circuits in both MDD and BD^4–7^. A meta-analysis of voxel-based morphometry (VBM) demonstrated that common and distinct patterns of grey-matter volume changes in MDD and BD, such as the prefrontal cortex, insula, and limbic system^7^. However, findings from task-based functional magnetic resonance imaging (fMRI) studies may be confounded by differences in the experimental design of the studies and in the tasks themselves^8^. Resting-state fMRI (rs-fMRI) provides a noninvasive and task-free approach that removes some performance-related confounds, and provides a reliable measure of ‘baseline’ brain activity and connectivity^9^. The amplitude of low frequency fluctuations (ALFF), an index to measure changes in resting-state blood oxygen level dependent (BOLD) signals, reflects cyclic modulation of gross cortical excitability and long distance neuronal synchronization^10,11^. It’s a relative and indirect measure of spontaneous brain activity. The current ALFF might be the most comparable measure to the resting positron emission tomography (PET). Both PET and ALFF measure the unconstrained, baseline state of mental activity. PET measures an averaged level of cerebral blood flow (CBF) or oxygen metabolism across a period of time, whereas ALFF measures the deviation, rather than the mean, of the BOLD signal^10^. The ALFF index was found to be stable, reliable and useful in characterizing the intrinsic or spontaneous brain activity in various brain diseases, such as Parkinson’s disease^12^, posttraumatic stress disorder^13^, MDD^14^, and BD^15^. Several previous studies have employed ALFF to investigate the pathophysiology of affective disorders, and found widespread aberrant regional spontaneous brain activity, including the medial prefrontal cortex (mPFC)^16^, precuneus/posterior cingulate cortex (PCC)^15,16^, temporal gyrus^17^, occipital gyrus^15,18^, cerebellum^19^, insula^20,21^, and limbic regions^21,22^. However, these results are often inconsistent, probably due to the small sample sizes, age ranges, clinical heterogeneity, and different methodology. Furthermore, it is at present unclear to what extent specific or common spontaneous brain activity alterations occur in MDD and BD given the paucity of direct comparisons.

The aim of this study was, therefore, to perform a quantitative and voxel-based meta-analysis of ALFF changes in MDD and BD, by taking advantage of the larger number of whole-brain rs-fMRI studies published in recent years. This is expected not only to enable a more precise understanding of the common and distinct pathophysiology of MDD and BD, but also to contribute to the identification of potential biomarkers for prevention and intervention in affective disorders.

## Methods

### Data sources, study selection, and quality assessment

A comprehensive search of studies published between January 1st, 2000 and April 9, 2019 was conducted in the PubMed, Embase, Web of Science, SinoMed, Chinese National Knowledge Infrastructure (CNKI), and WanFang databases using the keywords: (1) “depression” OR “depressive disorder” OR “major depression” OR “major depressive disorder” OR “depressed” OR “unipolar disorder”; AND “amplitude of low frequency fluctuation” OR “ALFF” OR “low frequency fluctuation” OR “LFF” OR “amplitude of low frequency oscillation” OR “LFO”; (2) “Bipolar Disorder” OR “Bipolar Disorders” OR “Disorder, Bipolar” OR “Psychosis, Manic-Depressive” OR “Psychosis, Manic Depressive” OR “Manic-Depressive Psychosis” OR “Manic Depressive Psychosis” OR “Affective Psychosis, Bipolar” OR “Bipolar Affective Psychosis” OR “Psychoses, Bipolar Affective” OR “Psychosis, Bipolar Affective” OR “Psychoses, Manic-Depressive” OR “Manic-Depressive Psychoses” OR “Psychoses, Manic Depressive” OR “Mania” OR “Manias” OR “Manic State” OR “Manic States” OR “State, Manic” OR “States, Manic” OR “Depression, Bipolar” OR “Bipolar Depression” OR “Manic Disorder” OR “Disorder, Manic” OR “Manic Disorders”; AND “amplitude of low frequency fluctuation” OR “ALFF” OR “low frequency fluctuation” OR “LFF” OR “amplitude of low frequency oscillation” OR “LFO”. In addition, the references of the included studies and relevant review articles were checked for additional relevant studies.

Studies that satisfied the following conditions were included in the meta-analysis. (1) Patients had been diagnosed with BD or MDD between 18 and 60 years old; (2) ALFF comparison of patients with BD or MDD versus HCs was conducted. As we know, fractional ALFF (fALFF) approach, is the ratio of power spectrum of low-frequency (0.01–0.08 Hz) range to that of the entire frequency range (0–0.25 Hz). The approach of fALFF may effectively suppress non-specific signal components in the resting-state fMRI, and therefore would significantly improve the sensitivity and specificity in detecting regional spontaneous brain activity, but is not as stable as ALFF in gray matter regions^11,23^. Thus, we only focused on ALFF studies in this meta-analysis. (3) Three-dimensional coordinates (Talairach or Montreal Neurological Institute [MNI]) were reported for the whole-brain ALFF analysis. (4) Significant results were reported using thresholds for significance corrected for multiple comparisons or uncorrected with spatial extent thresholds. And (5) the study was published as an original article (not as a letter or an abstract) in a peer-reviewed English or Chinese language journal.

Datasets were excluded if (1) patients with MDD/BD diagnosed with comorbid neurological or psychiatric diseases; (2) the data were unavailable (e.g., missing neuroanatomical coordinates) even after the authors were contacted by email or telephone; (3) the data overlapped with those of another included publication; (4) a region-of-interest approach was used.

We used a 10-point checklist involved in previous meta-analysis of rs-fMRI studies to assess the quality of each study selected for this meta-analysis (Table S1 in Supplementary materials)^24,25^. Literature search, study evaluation, and selection were independently performed by three investigators (G.J.Y., Q.S.J., and C.P.). Any discrepancies were resolved by a fourth investigator (W.Y.) for a final decision. The current study was conducted with reference to the Meta-analysis of Observational Studies in Epidemiology (MOOSE) guidelines for the meta-analyses of observational studies^26^.

### Voxel-wise meta-analysis

A meta-analysis of ALFF differences between patients and HCs was conducted for MDD and BD separately using the seed-based *d* mapping (SDM) software package (version 5.15 for Windows) in a standard process (www.sdmproject.com). The SDM approach uses effect sizes to combine reported peak coordinates that are extracted from databases with statistical parametric maps, and it recreates original maps of the effect size of ALFF difference between patients and HCs. We performed the analysis as described in the SDM tutorial and related publications and used MRIcron software (www.mricro.com/mricron/) to visualize SDM maps.

The SDM approach was briefly described here. We first extracted peak coordinates and effect size (e.g., *t*-values) of differences in ALFF between patients and HCs from each dataset. A standard MNI map of the ALFF differences was then separately recreated for each dataset using an anisotropic Gaussian kernel. The mean map was finally generated by voxel-wise calculation of the random-effects mean of the dataset maps, weighted by the sample size, intra-dataset variability, and between-dataset heterogeneity. To optimally balance false positives and negatives, we used the default SDM kernel size and thresholds (full width at half maximum (FWHM) = 20 mm, *p* = 0.005, uncorrected for false discovery rate (FDR), peak height *Z* = 1, cluster extent = 10 voxels)^27,28^. It should be noted that this FWHM kernel is intended to assign indicators of proximity to reported coordinates but not to smooth any image that is different in nature.

Next, a quantitative meta-analytic comparison of altered ALFF between MDD and BD was conducted by calculating differences between the two groups in each voxel and the statistical significance was determined using a standard randomization test^29,30^. During this process, we included age (i.e., mean age) and sex (i.e., sex distribution) as covariates. The meta-analysis used the default kernel size and thresholds in SDM to optimize sensitivity while controlling false positives: *p* < 0.005 with peak height *Z* > 1 and a cluster extent of >10 voxels^28,31^.

In addition, the overlap of ALFF-decrease/increase between MDD and BD via conjunction of thresholded meta-analytic results-maps was investigated. To test the reliability of the overlapping approach and to provide a mean statistical significance, we performed a Monto Carlo simulation with 5000 iterations. In each iteration step, we generated two Gaussian distribution brain maps within the 20% GMV mask based on the statistical mean and standard deviation of the *t*-maps of the MDD and BD. We first conducted an FDR correction (*p* < 0.05) for each of the two simulated maps and then overlapped the two maps in MNI space. Next, we determined the intersection regions and recorded the number of voxels within these regions. Finally, we compared the number of voxels in the actual interaction regions with these in the simulated data with a null distribution.

In addition, we repeated the meta-analyses above in subgroups, i.e., in unmedicated and depressed subgroup patients, respectively. We did not do the same analyses in other subgroups for their limited number of studies.

### Jackknife sensitivity analysis

Following preprocessing of the data, a whole-brain voxel-based jackknife sensitivity analysis was performed to test the robustness of the findings by iteratively repeating the same analysis, excluding one dataset each time^28^. This analysis is to establish the extent to which the results could be replicated. If a brain region remained significant in all or most of (>50%) the combinations of studies, we considered the finding to be highly replicable^14,32^.

### Analysis of heterogeneity and publication bias

A heterogeneity analysis was conducted using a random effects model with *Q* statistics to explore unexplained between-study variability in the results. Heterogeneous brain regions were obtained using the default SDM kernel size and thresholds (FWHM = 20 mm, *p* = 0.005, uncorrected for FDR, peak height *Z* = 1, cluster extent = 10 voxels)^27,28^.

In addition, Egger’s test was performed using the Stata/SE 12.0 software for Windows (Stata Corp LP, College Station, TX, USA) to assess possible publication bias by extracting the values from statistically significant relevant peaks between patients and HCs^28^. A *p*-value less than 0.05 was considered significant.

### Meta-regression analyses

Meta-regression analyses were carried out to examine the effects of clinical variables (e.g., illness duration, Hamilton depression rating scale [HAMD] score, Young mania rating scale score [YMRS]), which could potentially influence the analytic results. The results were weighted by the square root of the sample size. To minimize the reporting of spurious relationship, we selected a more conservative threshold of *p* = 0.0005 as used in previous studies^28^, requiring abnormalities to be detected both in the slope and in one of the extremes of the regressor, and discarding findings in regions other than those detected in the main analyses.

## Results

### Included studies

**Fig. 1:**
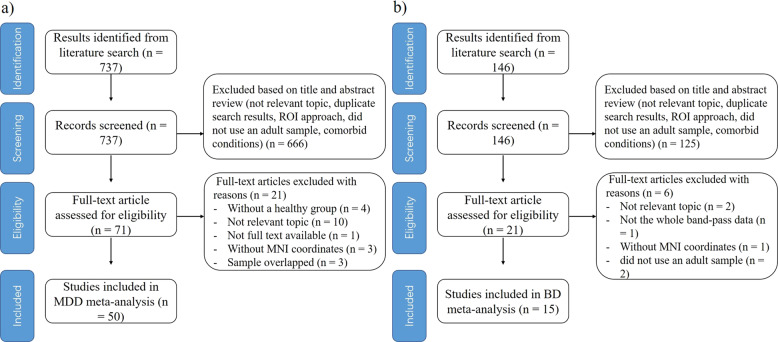
The flowchart describing the process of study inclusion. Flow chart of MDD meta-analysis study selection (**a**) and BD meta-analysis study selection (**b**). MDD major depressive disorder, BD bipolar disorder.

The first research strategy generated 737 matches. After initially removing the duplicates and reviewing the titles and abstracts, 66 studies were identified as potentially eligible for inclusion. After a detailed review of the full article text, 21 studies were excluded. Finally, 45 studies reporting 51 datasets that investigated ALFF differences between patients with MDD and HCs were eligible for inclusion in the meta-analysis. The second research strategy generated 146 matches. After initially removing the duplicates and reviewing the titles and abstracts, 16 studies were identified as potentially eligible for inclusion. After a detailed review of the full article text, 6 studies were excluded. Finally, 10 studies investigated ALFF differences between patients with BD and HCs were eligible for inclusion in the meta-analysis. Five studies compared both MDD and BD with control subjects. To sum up, we included 50 studies on MDD and 15 studies on BD. Among them, patients in 33 of 50 studies on MDD were unmedicated, and in 46 studies were in depressed state; while patients in 3 of 15 studies on BD were unmedicated, and in 6 studies were in depressed state. A flow diagram of the identification and exclusion of studies was presented in Fig. 1.

### Sample characteristics

#### MDD

The included datasets reported ALFF differences between 1399 patients with MDD (600 males and 799 females; mean age = 33.2 years; mean illness duration = 11.9 months) and 1332 HCs (608 males and 724 females; mean age = 32.9 years). No significant differences were observed between patients with MDD and HCs with respect to age (standardized mean difference [SMD] = ∼0; 95% confidence interval [CI] = −4.222 to 4.976, *t* = 0.163, *p* = 0.871) or sex distribution (*χ*^2^ = 2.104, *p* = 0.154). The mean score from the Hamilton depression rating scale (HAMD) was 23.1.

#### BD

The included datasets reported ALFF differences between 494 patients with BD (243 males and 251 females; mean age = 29.2 years; mean illness duration = 36.8 months) and 593 HCs (270 males and 323 females; mean age = 29.8 years). Of the 494 patients, 210 (42.5%) were depressed at the time of scanning, 73 (14.8%) were euthymic, 58 (11.7%) were manic, 3 (0.6%) were mixed, and 1 (0.2%) was hypomanic; mood state was not available for 149 patients (30.2%). In relation to bipolar subtypes, 95 patients were type I and 128 patients were type II; the subtype was not available in 271 patients. No significant differences were observed between patients with BD and HCs with respect to age (SMD = ∼0; 95% CI = −4.314 to 3.200, *t* = −0.305, *p* = 0.763) or sex distribution (*χ*^2^ = 3.563, *p* = 0.066). The mean score from the HAMD was 16.4 and mean YMRS score was 9.6.

#### MDD versus BD

MDD and BD patients were not similar with respect to sex (*χ*^2^ = 9.566, *p* = 0.002) and mean age (*t* = 2.545, *p* = 0.013). Duration of illness was greater in BD (36.8 months vs. 11.9 months, *p* < 0.05). Predictably, the proportion of depressed patients was higher in the MDD group (92% vs. 43%).

The demographic, clinical, imaging characteristics, and quality scores of the included studies in this meta-analysis were well described in Table S2 in supplementary materials.

### ALFF differences of the main meta-analysis

#### MDD versus HCs

**Fig. 2:**
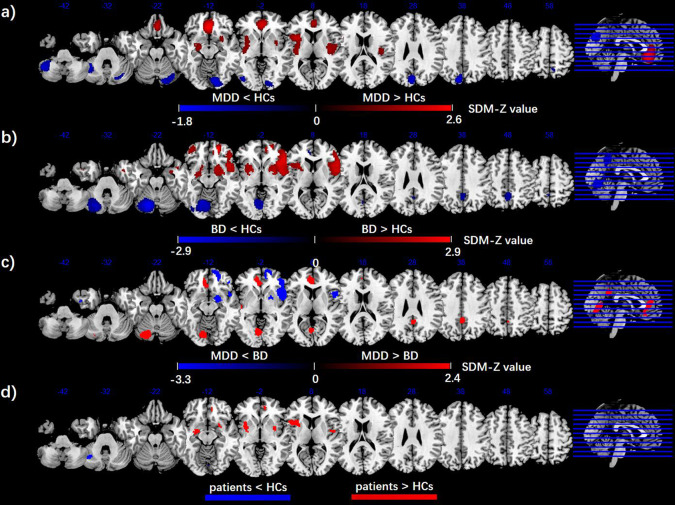
Brain regions showed significant ALFF differences between groups based on the meta-analyses. Meta-analyses results regarding **a** ALFF difference between MDD and HCs, **b** ALFF difference BD and HCs, **c** ALFF difference between MDD and BD (vs. HCs), as well as **d** conjunction of MDD and BD (vs. HCs). Areas with decreased ALFF value are displayed in blue, and areas with increased ALFF value are displayed in red. The color bar indicates the maximum and minimum SDM-Z values. HCs healthy controls, MDD major depressive disorder, BD bipolar disorder, SDM seed-based *d* mapping.

As illustrated in Fig. 2a, the meta-analytic brain map showed both decreased and increased ALFF in MDD patients relative to HCs. Patients with MDD displayed increased ALFF in the right superior frontal gyrus (SFG) (including the medial orbitofrontal cortex, mPFC, and anterior cingulate cortex [ACC]), bilateral insula extending into the striatum, and left supramarginal gyrus, and decreased ALFF in the bilateral posterior lobes of cerebellum, bilateral precuneus, and left occipital cortex compared with HCs. These areas did not show significant between-study heterogeneity (all *p* values > 0.05). Egger’s tests of publication bias were nonsignificant except in the left cerebellum and occipital gyrus (*p* < 0.05). A jackknife sensitivity analysis revealed that in MDD patients, the most robust data were obtained for increases in ALFF in the right SFG, left insula, and left supramarginal gyrus and decreases in ALFF in the bilateral cerebellum, and left precuneus, replicable in all 56 datasets. The increased ALFF in the right insula and decreased ALFF in the left occipital gyrus and right precuneus remained replicable, as they were significant in at least 54/56 of the datasets. The results from the SDM analysis were summarized in Table 1.

**Table 1:** Meta-analyses results regarding ALFF difference between MDD and HCs, BD and HCs, as well as between MDD and BD (vs. HCs), respectively.

Local maximum				Cluster		Egger’s test (*p* value)	Jackknife sensitivity	Heterogeneity
Region	Peak MNI coordinate (x, y, z)	SDM-Z value	*p* value	No. of voxels	Breakdown (No. of voxels)			
*MDD vs. HCs (*MDD > HCs*)*								
Right superior frontal gyrus, medial orbital, BA 11	4, 36, −12	2.56	5.19e−6	1970	Left superior frontal gyrus, BA 10 (229) Right anterior cingulate/paracingulate gyri, BA 11 (235) Left anterior cingulate/paracingulate gyri, BA 10 (380)	0.158	56/56	No
Left insula	−36, −16, 4	1.62	4.80e−4	1724	Left putamen, BA 48 (217) Left heschl gyrus, BA 48 (90) Left superior longitudinal fasciculus III (89) Left inferior frontal gyrus, BA 48 (99) Left striatum (53) Left hippocampus (99) Left amygdala, BA 34 (23)	0.902	56/56	No
Right insula, BA 48	36, −14, 6	1.64	4.44e−4	1033	Right putamen, BA 48 (163) Right rolandic operculum, BA 48 (139) Right heschl gyrus, BA 48 (120)	0.896	55/56	No
Left supramarginal gyrus, BA 48	−56, −40, 24	1.56	6.50e−4	240	Left superior temporal gyrus, BAs 42, 48 (116)	0.680	56/56	No
*MDD vs. HCs (MDD* < *HCs)*								
Right cerebellum, hemispheric lobule VI, BA 18	16, −84, −16	−1.74	1.03e−5	1495	Right lingual gyrus, BA 18 (393) Right inferior network, inferior longitudinal fasciculus (146) Right fusiform gyrus, BA 19 (86) Right calcarine fissure/surrounding cortex, BA 18 (81) Right inferior occipital gyrus, BA 18 (107)	0.725	56/56	No
Left cerebellum, crus I	−42, −54, −34	−1.53	7.22e−5	1035		0.015	56/56	No
Left cuneus cortex, BA 19	−6, −82, 34	−1.64	2.06e−5	630	Left precuneus, BA 7 (79) Corpus callosum (54) Left superior occipital gyrus, BA 19 (25)	0.948	56/56	No
Left inferior network, inferior longitudinal fasciculus	−36, −78, 2	−1.32	5.37e−4	178	Left middle occipital gyrus, BAs 18, 19 (106) Left inferior occipital gyrus, BA 19 (28)	0.024	54/56	No
Right precuneus, BA 5	6, −58, 58	−1.11	2.40e−4	32		0.533	54/56	No
*BD vs. HCs (*BD > HCs*)*								
Right inferior frontal gyrus, orbital part, BA 47	48, 28, −4	2.63	<e−10	4243	Right insula, BAs 47, 48 (810) Right striatum (280) Right putamen, BA 48 (237) Right rolandic operculum, BA 48 (204) Right temporal pole, superior temporal gyrus, BA 38 (214) Right superior longitudinal fasciculus III (120) Right amygdala, BA 34 (116) Right superior temporal gyrus, BA 48 (68)	0.164	15/15	No
Left insula, BA 48	−32, 14, 6	2.18	6.19e−5	1114	Left striatum (220) Left putamen (214) Left superior longitudinal fasciculus III (63) Left inferior frontal gyrus, BAs 45, 48 (71) Left amygdala, BA 34 (24)	0.678	15/15	No
Right superior frontal gyrus, orbital prefrontal gyrus, BA 11	22, 50, −12	2.85	<e−10	305	Right middle frontal gyrus, BA 11 (51) Right striatum (27)	0.881	15/15	Yes
Left inferior frontal gyrus, orbital part, BA 47	−38, 48, −12	1.81	9.65e−4	257		0.451	13/15	No
Right superior frontal gyrus, medial orbital, BA 10	12, 48, −6	2.32	1.03e−5	139		0.665	15/15	Yes
Right superior frontal gyrus, dorsolateral, BA 8	20, 14, 54	1.63	2.75e−3	67		0.238	13/15	No
Right inferior network, inferior longitudinal fasciculus	36, 0, −30	1.68	2.19e−3	34		0.533	15/15	No
*BD vs. HCs (BD* < *HCs)*								
Left cerebellum, hemispheric lobule VI, BA 18	−18, −70, −22	−2.85	1.03e−5	3628	Left lingual gyrus, BA 18 (553) Left fusiform gyrus, BA 18 (224) Left calcarine fissure/surrounding cortex, BA 17 (103) Left inferior occipital gyrus, BAs 18, 19 (29) Right lingual gyrus, BA 17 (42)	0.388	15/15	No
Right precuneus	2, −52, 48	−1.90	2.53e−4	568	Left precuneus (325)	0.301	15/15	No
Left anterior cingulate / paracingulate gyri, BA 32	−10, 44, 8	−1.42	3.30e−3	69		0.938	14/15	No
Left superior temporal gyrus, BA 48	−52, −20, 12	−1.44	3.04e−3	59		0.366	12/15	No
*MDD* > *HCs vs. BD* > *HCs (MDD* > *BD)*								
Left lingual gyrus, BA 17	−4, −74, −4	−2.37	2.17e−4	1283	Left cerebellum, BAs 17 18 19 (628) Left fusiform gyurs, BAs 18 19 (69)	0.526	/71	No
Left median network, cingulum	−8, 40, −2	−2.38	2.17e−4	773	Left anterior cingulate/paracingulate gyri, BAs 10 11 32 24 25 (420) Left superior frontal gyrus, BAs 10 11 32 (136) Corpus callosum (86) Left gyrus rectus, BA 11 (22)	0.220	/71	No
Right median cingulate / paracingulate gyri, BA 23	4, −48, 32	−1.71	1.78e−3	253	Right precuneus (50) Left precuneus (28) Right posterior cingulate gyrus, BA 23 (33) Left posterior cingulate gyrus, BA 23 (45)	0.170	/71	No
Left superior temporal gyrus, BA 48	−42, −18, −4	−1.52	3.62e−3	36		0.605	/71	No
*MDD* > *HCs vs. BD* > *HCs (MDD* < *BD)*							/71	No
Right insula, BA 48	44, 14, −2	3.16	<e−10	1286	Right inferior frontal gyrus, BAs 45 47 48 (459) Right superior longitudinal fasciculus III (64) Right rolandic operculum, BA 48 (60) Right anterior thalamic projections (27) Right temporal pole (53)	0.560	/71	No
Right superior frontal gyrus, medial orbital, BA 10	12, 50, −6	3.22	<e−10	459	Corpus callosum (83) Right striatum (26) Right middle frontal gyrus, BA 11 (26)	0.804	/71	Yes
Right striatum	20, 2, −8	2.04	5.78e−4	318	Right lenticular nucleus, putamen, BA 48 (39) Right amygdala, BA 34 (31)	0.927	/71	No
Right frontal superior longitudinal	20, 14, 52	1.58	3.07e−3	23		0.749	/71	No
Right fusiform gyrus, BA 20	36, −4, −42	1.54	3.54e−3	20		0.363	/71/	No
Left striatum	−18, 6, 0	1.67	2.24e−3	16		0.212	/71	No

*HCs* healthy controls, *MDD* major depressive disorder, *BD* bipolar disorder, *MNI* Montreal Neurological Institute, *SDM* seed-based d mapping, *BA* Brodmann area.

#### BD versus HCs

As illustrated in Fig. 2b, the meta-analytic brain map showed both decreased and increased ALFF in BD patients relative to HCs. Patients with BD displayed increased ALFF in the bilateral inferior frontal gyrus (IFG) (including the orbital prefrontal cortex), bilateral insula extending into the striatum, right SFG (including the mPFC and dorsolateral prefrontal cortex), and right temporal pole, and decreased ALFF in the left posterior lobes of cerebellum (extending to the occipital cortex), bilateral precuneus, left ACC, and left superior temporal gyrus (STG). These areas did not show significant between-study heterogeneity except the right mPFC (*p* < 0.05). Egger’s tests of publication bias were nonsignificant (all *p* values > 0.05). A jackknife sensitivity analysis revealed that in BD patients, the most robust data were obtained for increases in ALFF in the right IFG, left insula, right SFG (orbital prefrontal gyrus), and right occipital gyrus and decreases in ALFF in the left cerebellum, and right precuneus, replicable in all 15 datasets. The increased ALFF in the left IFG, and right SFG (orbital part) and decreased ALFF in the left ACC and left STG remained replicable, as they were significant in at least 12/15 of the datasets (Table 1).

#### (MDD vs. HCs) versus (BD vs. HCs)

As illustrated in Fig. 2c, the meta-analytic brain map showed both decreased and increased ALFF in MDD patients relative to BD patients. MDD displayed increased ALFF in the left lingual gyrus, left ACC, bilateral precuneus/PCC, and left STG, and decreased ALFF in the right insula, right mPFC, right fusiform gyrus, and bilateral striatum relative to BD patients (Table 1).

#### (MDD vs. HCs) and (BD vs. HCs) conjunction

As illustrated in Fig. 2d, the conjunction analysis revealed that both MDD and BD had increased ALFF in bilateral insula, mPFC, and decreased ALFF in the left posterior lobe of cerebellum.

#### Meta-regression analyses

In patients with MDD, meta-regression analysis indicated that higher HAMD score was correlated with greater increases in ALFF in the right ACC. In patients with BD, meta-regression analysis revealed that higher HAMD score exhibited larger decreases in ALFF in the right PCC. Mean illness duration was not associated with any MDD or BD-related ALFF changes. The results of the meta-regression analyses were presented in Table 2.

**Table 2:** Results of meta-regression analyses for MDD and BD.

Group	Region	Peak MNI coordinate (x, y, z)	No. of voxels	SDM-Z value	*p* value
*MDD*	*Effect of HAMD score*				
	Abnormal activities in studies with higher HAMD score				
	Right anterior cingulate/paracingulate gyri, BA 32	6, 44, 28	32	1.70	2.27e−4
	Right median cingulate/paracingulate gyri, BA 23	4, −36, 36	123	−1.64	1.34e−4
*BD*	*Effect of illness duration*				
	Abnormal activities in studies with longer illness duration				
	Right anterior thalamic projections	16, 2, 16	131	2.51	2.58e−5
	Right striatum	28, −4, −6	20	−2.46	1.86e−4
	*Effect of HAMD score*				
	Abnormal activities in studies with higher HAMD score				
	Right posterior cingulate gyrus, BA 23	2, −50, 30	303	−2.98	<e−10
	*Effect of YMRS score*				
	Abnormal activities in studies with higher YMRS score				
	Right insula, BA 48	42, 10, −2	1133	5.38	<e−10
	Right inferior frontal gyrus, orbital part, BA 47	40, 44, −2	345	3.44	7.74e−5
	Left insula, BA 48	−36, 14, 8	231	4.34	<e−10
	Right amygdala, BA 34	22, −2, −10	67	2.87	4.23e−4
	Right superior frontal gyrus, BA 10	14, 46, 0	46	3.92	5.19e−6
	Left cerebellum	−26, −80, −24	839	−4.41	<e−10
	Right precuneus, BA 5	4, −52, 58	325	−3.11	1.03e−4

*MDD* major depressive disorder, *BD* bipolar disorder, *HAMD* Hamilton depression scale, *YMRS* Young Manic Rating Scale, *BA* Brodmann area, *MNI* Montreal Neurological Institute, *SDM* seed-based d mapping.

In addition, the results for meta-analyses on unmedicated subgroups were provided in Fig. S1 and Table S3, while the results on depressed subgroups were provided in Fig. S2 and Table S4 in the Supplementary materials. The main results remained largely unchanged.

## Discussion

In this paper, we report findings from the largest voxel-based meta-analysis conducted to date of ALFF studies in MDD and BD. We compared results to identify both common and different patterns of spontaneous brain activity alterations. MDD and BD shared increased ALFF in the bilateral insula and right mPFC, and decreased ALFF in the left cerebellum posterior lobe, suggesting that altered intrinsic activity in these regions is common to both disorders. Several regions, including the limbic system and occipital cortex, differed between conditions, indicating that these disorders may be associated with spatially distinct patterns of brain function.

The insula is a cortical structure with extensive connections to many areas of the cortex and limbic system, which implicated in disparate cognitive, affective, and regulatory functions, including interoceptive awareness, emotional responses, and empathic processes^33^. We found increased ALFF in the bilateral insula (extending into the striatum) in MDD and BD, suggesting a consistently abnormal pattern of intrinsic activity in both disorders. Furthermore, increasing ALFF of the right insula was significantly greater in BD than MDD. A recent multimodal meta-analysis^34^ noted increased ALFF and regional CBF (rCBF) in the left insula in MDD. Of note, this meta-analysis included eight ALFF studies and eight rCBF studies. More recently, several rs-fMRI studies found altered functional connectivity between the insula-centric salience network (SN) and the default mode network (DMN), and the central executive network (CEN) in MDD and BD^35,36^. The insula-mediated dynamic switching between the DMN and the CEN facilitates access to cognitive resources, such as attention and working memory, when a salient event is detected. Thus, altered strength in the connectivity in these networks affects cognitive deficits in some cases of affective disorders^37^. Previous task-based fMRI studies found abnormal activity of the insula during executive functioning tasks and emotional processing tasks in MDD^38^ and BD^39^, which may partly explain the difficulties in cognitive and emotional integration in affective disorders. Moreover, changes in insula activity occur with a variety of treatments for depression, including medication, deep brain stimulation, and cognitive behavior therapy, suggesting a role for this region in mediating antidepressant response and remission more generally^40^. Several meta-analysis of structural morphometric studies also found reduced grey-matter volume in the bilateral insula in MDD and BD^7,35,41^. Thus, functional and structural abnormality of the insula may be a key neurobiological feature of affective disorders.

The prefrontal cortex is considered as a key neuronal region in regulating attention, cognitive control, motivation, and emotion^42^. We found increased ALFF in the prefrontal cortex in MDD and BD. In MDD, these alterations were predominantly located in the most bilateral SFG, including the mPFC, medial orbitofrontal cortex, and ACC, while in BD these alterations were located in the bilateral IFG (including the orbital prefrontal cortex) and right SFG (including the mPFC and dorsolateral PFC). The conjunction analysis indicated that ALFF of the right mPFC was robustly higher in both conditions, suggesting a consistent intrinsic activity abnormal pattern across disorders. A recent meta-analysis confirmed the association between rumination and DMN core regions- the mPFC subsystem activation. Based on those findings, they suggested a hypothesis of how DMN regions support rumination and presented the implications of this model for treating MDD characterized by rumination^43^. Several studies showed that people who engaged in ruminative responses to depressive symptoms had higher levels of depressive symptoms over time, after accounting for baseline levels of depressive symptoms^44^. Another meta-analysis of VBM study reported that both MDD and BD shared similar patterns of lower grey-matter volume in the mPFC^7^. These findings of the mPFC, in conjunction with increased ALFF in the bilateral insula findings, suggest the increased intrinsic activity of the mPFC and insula could be a compensatory response to structural deficits in both MDD and BD. Several proton magnetic resonance spectroscopy (^1^H-MRS) studies found abnormal levels of glutamate + glutamine (Glx) within the prefrontal cortex in MDD^45^ and BD^46^. Glutamate is the major excitatory neurotransmitter in the cerebral cortex. A combined fMRI-MRS study found glutamate concentration in the mPFC correlated with the ALFF in the same area of the MDD patients, suggesting a unique functional-metabolic coupling^47^. Glutamate could coordinate both the vascular and metabolic responses to neuronal activity underlying functional imaging signal changes^48,49^. Post-mortem studies also noted increased levels of glutamate in the prefrontal cortex of both MDD and BD^50^, suggesting a perturbed frontal glutamate system. Taken together, these observed changes of the prefrontal cortex possibly lead to abnormalities in cognition, behavior, and emotion in affective disorders.

We found decreased ALFF in the left posterior lobes of cerebellum (mainly in lobule VI and crus I) in MDD and BD, and decreased ALFF in the right posterior lobes of cerebellum (mainly in lobule VI) in MDD, suggesting disrupted intrinsic activity of the posterior cerebellum in both disorders. A previous meta-analysis of eight ALFF studies demonstrated decreased ALFF in the cerebellum in medication naïve patients with MDD^14^. The anterior hemisphere of the cerebellum is regarded as being primarily related to motor learning and coordination while the posterior lobe is involved in emotion, awareness, and cognitive processing of higher-order functions in humans^51^. There is increasing evidence that the cerebellum is connected to cortical areas involved in the pathophysiology of psychiatric disorders^52–54^. Abnormal structure and function in the cerebellum (especially in the cerebellar posterior lobules VI, VIIa (Crus I), IX, and in the posterior area of the vermis)^55^ have been reported in both BD and MDD patients, including abnormalities of gray matter volume^56,57^, glucose metabolism^58,59^, functional activity, and connectivity^6,12,18,52,60–62^. Moreover, dysregulation between the limbic cerebellum and the well-known limbic cerebral networks consequent to a cerebellar lesion is at the root of BD, at least the manic state, and provides a new framework for interpreting cerebellar modulation in the regulation of mood in specific psychiatric conditions^63^. Cerebellar dysfunction might slow the data integration necessary for mood state awareness, resulting in difficulty of depressed cerebellar damage patients in explicitly recognizing their mood^64^. Therefore, our findings provide additional evidence for the involvement of cerebellar dysfunction in the pathophysiology of affective disorders.

In addition, we found decreased ALFF in the left occipital cortex in MDD and decreased ALFF in the left cerebellum (extending to the occipital cortex) in BD. Furthermore, BD showed lower ALFF in the left lingual gyrus than MDD. The occipital lobe contains most of the anatomical region of the visual cortex and contributes to visual information processing and communication with the cerebral cortex, and plays a role in the perception of facial emotion^65^. Few studies have reported the occipital lobe changes in mood disorders. However, some studies found structural^5,7,66^, functional^15,65,67,68^, and metabolic^69^ abnormalities in the occipital cortex in MDD and BD. Moreover, several researches considered the model of selective bias in processing and interpretation for emotional information to be one of the risk factors of MDD for young people and that attention to negative information may maintain depression^70–73^. Taken together, these findings of disrupted intrinsic activity in the occipital cortex suggest that processing bias in affective disorders may be initiated as a perceptual visual bias, which may cause a series of cognitive and affective symptoms.

MDD and BD showed different ALFF changes, most prominently in the left ACC, left occipital cortex, right PCC, right insula and right medial orbitofrontal cortex. Furthermore, our findings of increased ALFF in the ACC are specific to MDD, and decreased ALFF in the PCC/ precuneus is specific to BD, suggesting that a differential pattern of intrinsic activity in the limbic system may potentially differentiate these two disorders. Meta-regression analysis demonstrated that higher HAMD scores were associated with greater increase of ALFF in the ACC in MDD, and were associated with greater decrease of ALFF in the PCC in BD. Therefore, altered intrinsic activity of the cingulate cortex is very likely a state effect. The cingulate cortex is an important interface between emotional regulation, sensing and action. Previous task-based fMRI studies found abnormal ACC activation during the facial emotional processing^74^ and executive control paradigm^75^ in MDD but not in BD, potentially indicating different pathophysiologic processes, especially in emotion regulation and attentional control neural circuitry in MDD versus BD^76^. In addition, activation patterns in the ACC, especially the subgenual ACC, have been shown to successfully predict treatment response for antidepressant medication and electroconvulsive therapy^77^. However, several studies found decreased glial density^78^, gray matter volume^7^, and cortical thickness^79,80^ in the ACC in MDD and BD. The relationship between functional and structural alterations in these conditions remains unclear; and further research is essential to understand potential functional and/or structural disease-specific alterations within affective disorders in the limbic system.

## Limitations

This meta-analysis had some limitations. First, we could not determine whether these functional alterations were part of the pathogenesis or a consequence of these disorders because of the nature of cross-sectional studies. Second, the samples used in the studies differed between disorders with respect to treatment status. Given that psychotropic medications can have effects on brain function, it is difficult to be certain that results are not entirely independent from medication status. However, because the subgroup analysis of drug-naive MDD patients yielded similar findings as data from the whole group analysis (Fig. S1 and Table S3), the effects we observed to seem most likely to be illness related rather than treatment-related. A further limitation is the difference in mood states of MDD and BD patients. The majority of patients with MDD were currently depressed at the time of scanning, while the majority of BD participants were euthymic or depressed, and the limited number of studies in each category prevented statistical analysis of mood state effects. Third, clinical details were often insufficiently reported in the studies for comprehensive and powerful subgroup or meta regression analyses. Including a range of clinical variables in future neuroimaging studies would benefit future meta analyses greatly. Relevant clinical information could include bipolar subtype (e.g., I/II), mental state at the time of scanning (e.g., depressive, manic, and euthymic phase), rating scale scores, medication status, type of medication administered (e.g., antidepressant, mood stabilizer and antipsychotics), and duration of administration, details of comorbidities, age of onset/duration of illness, and number of episodes. Finally, age and sex were significantly different between MDD and BD patients in this study. But we included age and sex as covariates in conducting a quantitative meta-analytic comparison of altered ALFF between MDD and BD.

## Conclusion

In conclusion, the current meta-analysis demonstrates that MDD and BD show a common pattern of aberrant regional intrinsic brain activity, which predominantly includes the insula, mPFC, and cerebellum. In addition, the two conditions also show distinct patterns of brain activity alterations in the limbic system regions (particularly in the cingulate cortex) and occipital cortex. These findings seem to imply that MDD and BD have more brain functionally similar than they are different. These results expand on a growing literature exploring resting-state activity in MDD and BD, which provide useful insights for understanding the underlying pathophysiology of brain dysfunction in affective disorders, and developing more targeted and efficacious treatment and intervention strategies.

## Supplementary information

Supplementary material
