# Supplementary material for: Common and distinct patterns of intrinsic brain activity alterations in major depression and bipolar disorder: voxel-based meta-analysis
Source: Transl Psychiatry. 2020 Oct 19;10:353. doi: 10.1038/s41398-020-01036-5 (PMC7573621; doi:10.1038/s41398-020-01036-5)
Supplement: Supplementary file 1 — Supplementary material [file 41398_2020_1036_MOESM1_ESM.doc]

**Common and distinct patterns of intrinsic brain activity alterations in** **major depression and bipolar disorder: voxel-based meta-analysis**

**Table S1 Quality assessment checklist (score 0/0.5/1 per item; total score out of 10)***

| Category 1: Participants |
| --- |
| 1. Patients were evaluated prospectively, specific diagnostic criteria were applied, and demographic data were reported. |
| 2. Healthy comparison participants were evaluated prospectively, psychiatric and medical illnesses were excluded. |
| 3. Important variables (e.g., age, sex, illness duration, onset, medication status, comorbidity, severity of illness) were checked either by stratification or statistically. |
| 4. Sample size per group > 10. |
| Category 2: Methods for image acquisition and analysis |
| 5. Whole brain analysis was automated with no a priori regional selection. |
| 6. Coordinates reported in a standard space. |
| 7. The imaging technique used was clearly described so that it could be reproduced. |
| 8. Measurements were clearly described so that they could be reproduced. |
| Category 3: Results and conclusions |
| 9. Statistical parameters for significant and important nonsignificant differences were provided. |
| 10. Conclusions were consistent with the results obtained and the limitations were discussed. |
| *When criteria were partially met, 0.5 points were awarded. |
|  |

**Table S2. The demographic, clinical, and imaging characteristics of the included studies in this meta-analysis**

| **Study** | **Demographic characteristics** | | | | **Clinical characteristics: patients only** | | | | | **Imaging characteristics** | | | | **Quality**  **score^** |
| --- | --- | --- | --- | --- | --- | --- | --- | --- | --- | --- | --- | --- | --- | --- |
|  | Subjects (males), n | | Mean age, years | | Illness duration, months | HAMD-17 | Antidepressants (%) | State |  | Scanner | Software | FWHM, mm | Threshold |  |
| **Samples from MDD patients** | | | | | | | | | | | | | | |
|  | **MDD** | **HC** | **MDD** | **HC** |  |  |  |  |  |  |  |  |  |  |
| Bai et al., 2010^1^ | 49 (24) | 42 (20) | 29.9 | 27.6 | 10.8 | 22.4 | 0 | Depressed |  | 3.0 T | REST | 8 | *p* < 0.001 (uncorrected) | 10 |
| Ding et al., 2010^2^ | 18 (10) | 18 (10) | 15.8 | 16.2 | NA | NA | 0 | Depressed |  | 3.0 T | REST | 8 | *p* < 0.05（uncorrected） | 9 |
| Xu et al., 2010^3^ | 14 (8) | 14 (8) | 29.1 | 30.2 | NA | NA | 0 | Depressed |  | 3.0 T | SPM5 | 4 | *p* < 0.005（uncorrected） | 9 |
| Jiao et al., 2011^4^ | 18 (8) | 18 (8) | 15.8 | 16.2 | 6.0 | NA | 0 | Depressed |  | 3.0 T | REST | 4 | *p* < 0.05（Alphasim） | 9.5 |
| Wang et al., 2012^5^ | 18 (9) | 18 (9) | 34.0 | 35.0 | 5.0 | 25.0 | 0 | Depressed |  | 3.0 T | DPARSF | 4 | *p* < 0.05（Alphasim） | 10 |
| Zhu et al., 2012^6^ | 19 (10) | 18 (9) | 55.6 | 53.7 | 3.5 | 23.7 | 0 | Depressed |  | 1.5 T | REST | 8 | *p* < 0.05（Alphasim） | 10 |
| Fan et al., 2013^#7^ | 27 (11) | 56 (25) | 34.4 | 36.5 | 6.1 | 22.3 | 96 | Depressed |  | 3.0 T | REST | NA | *p* < 0.01（Alphasim） | 9.5 |
|  | 9 (4) |  | 38.4 |  | 11.6 | 20.9 | 33 | Depressed |  | 3.0 T |  |  |  | 10 |
| Guo et al., 2013a^8^ | 23 (12) | 23 (11) | 32.0 | 32.6 | NA | NA | 0 | Depressed |  | 3.0 T | REST | 4 | *p* < 0.005（uncorrected） | 9 |
| Guo et al., 2013b^#9^ | 15 (6) | 15 (8) | 24.1 | 23.9 | 3.3 | 22.9 | 0 | Depressed |  | 1.5 T | REST | 8 | *p* < 0.05 (FDR) | 9.5 |
|  | 15 (6) | 15 (6) | 67.5 | 64.9 | 5.9 | 21.6 | 0 | Depressed |  |  |  |  |  |  |
| Jing et al., 2013^#10^ | 19 (0) | 19 (0) | 34.8 | 36.8 | 6.9 | 21.7 | 84 | Depressed |  | 3.0 T | REST | 4 | *p* < 0.05（Alphasim） | 9.5 |
|  | 19 (0) |  | 37.6 |  | 7.4 | 4.6 | 95 | Remitted |  |  |  |  |  |  |
| Li et al., 2013^11^ | 30 (9) | 30 (12) | 29.4 | 32.4 | NA | NA | 0 | Depressed |  | 3.0 T | REST | 4 | *p* < 0.05（FDR） | 9 |
| Yang et al. 2013^12^ | 19 (9) | 19 (9) | 24.9 | 26.2 | 34.2 | 31.8 | 100 | Depressed |  | 3.0 T | DPARSF | 4 | *p* < 0.005（uncorrected） | 10 |
| Gong et al., 2014^13^ | 15 (10) | 16 (10) | 15.0 | 15.0 | NA | NA | 0 | Depressed |  | 3.0 T | REST | 4 | *p* < 0.005 (uncorrected) | 9 |
| Jia et al., 2014^14^ | 13 (7) | 14 (7) | 31.2 | 31.8 | NA | NA | 0 | Depressed |  | 3.0 T | DPARSF  REST | NA | *p* < 0.001 (Alphasim) | 8.5 |
| Jiang et al., 2014^15^ | 25 | 25 | 29.8 | 30.5 | NA | 26.2 | 0 | Depressed |  | 3.0 T | DPARSF | 6 | *P* < 0.05 (uncorrected) | 9 |
| Liu et al., 2014^16^ | 30 (13) | 30 (15) | 29.9 | 30.2 | 13.3 | 28.5 | 0 | Depressed |  | 3.0 T | REST | 4 | *p* < 0.05（Alphasim） | 10 |
| Qiu et al., 2014^17^ | 13 (3) | 14 (4) | 34.8 | 33.7 | NA | 40.6 | 0 | Depressed |  | 3.0 T | REST | 8 | *p* < 0.001 (FDR) | 9.5 |
| Yan et al., 2014a^18^ | 14 (0) | 18 (0) | 36.0 | 33.0 | 4.0 | 25.7 | 0 | Depressed |  | 3.0 T | DPARSF | 4 | *p* < 0.05（Alphasim） | 10 |
| Yan et al., 2014b^19^ | 22 (12) | 26 (12) | 34.0 | 32.0 | 4.9 | 25.7 | 0 | Depressed |  | 3.0 T | REST | 4 | *p* < 0.05（Alphasim） | 10 |
| Zhang et al., 2014^20^ | 32 (14) | 35 (18) | 20.5 | 21.0 | NA | NA | 0 | Depressed |  | 1.5 T | REST | 8 | *p* < 0.05 (FWE). | 9 |
| Zhao et al., 2014^21^ | 51 (24) | 50 (22) | 28.0 | 29.0 | NA | NA | 0 | Depressed |  | 3.0 T | REST | 8 | *p* < 0.001 (uncorrected) | 9 |
| Fang et al., 2015^22^ | 20 (12) | 18 (10) | 59.2 | 59.1 | 3.6 | 26.6 | 0 | Depressed |  | 1.5 T | SPM8, REST | 4 | *p* < 0.05, GRF correction | 9.5 |
| Li et al., 2015^23^ | 56 (27) | 22 (13) | 34.8 | 34.9 | NA | NA | NA | Depressed |  | 3.0 T | REST | 4 | *p* < 0.05（corrected） | 8.5 |
| Qiu et al., 2015^24^ | 5 (1) | 1 | 55~60^*^ | 55~60^*^ | NA | NA | 0 | Depressed |  | 3.0 T | REST | NA | *p* < 0.05（Alphasim） | 8 |
| Yong et al., 2015^25^ | 30 (15) | 30 (15) | 34.4 | 35.1 | NA | NA | 0 | Depressed |  | 3.0 T | SPM | 4 | *p* < 0.05 (Uncorrected) | 9 |
| Du et al., 2016^26^ | 18 (5) | 18 (10) | 39.3 | 35.3 | NA | NA | 0 | Depressed |  | 3.0 T | REST | 4 | *p* < 0.001（Alphasim） | 9 |
| Guo et al., 2016^27^ | 30 | 30 | 18~70^*^ | 18~70^*^ | NA | NA | 0 | Depressed |  | 3.0 T | REST | NA | *p* < 0.001（uncorrected） | 8.5 |
| Wang et al., 2016a^28^ | 35 (12) | 32 (12) | 33.6 | 33.7 | 5.1 | 27.1 | 0 | Depressed |  | 3.0 T | REST | 6 | *p* < 0.05（Alphasim） | 10 |
| Wang et al., 2016b^29^ | 18 (6) | 17 (5) | 27.7 | 30.2 | NA | 29.0 | 100 | Depressed |  | 3.0 T | DPARSF  DPABI | 6 | *p* < 0.005 (Alphasim) | 9.5 |
| Wei et al., 2016^30^ | 12 | 11 | 34.1 | 29.7 | NA | 24.5 | NA | NA |  | 3.0 T | DPARSF | 4 | *P* < 0.05 (Alphasim) | 8.5 |
| Zhang et al., 2016a^31^ | 32 (14) | 35 (18) | 20.5 | 21.0 | NA | NA | 0 | Depressed |  | 1.5 T | REST | 8 | *p* < 0.001 (uncorrected) | 9 |
| Zhang et al., 2016b^32^ | 11 (0) | 11 (0) | 34.1 | 33.6 | NA | 22.9 | 55 | Depressed |  | 3.0 T | REST | 4 | *p* < 0.01（Alphasim） | 9.5 |
| Zhao et al., 2016^33^ | 32 (0) | 40 (0) | 59.1 | 58.1 | NA | NA | 100 | Depressed |  | 3.0 T | DPARSF | 6 | *p* < 0.05（Alphasim） | 9 |
| Zhu et al., 2016^34^ | 27 (13) | 28 (14) | 21.7 | 21.3 | NA | NA | 0 | Depressed |  | 3.0 T | REST | 4 | *p* < 0.05（Alphasim） | 9 |
| He et al., 2017^35^ | 22 (13) | 22 | 37.8 | 35.1 | NA | 25.6 | NA | NA |  | 3.0 T | DPARSF | 4 | *P* < 0.01 (Alphasim corrected) | 8.5 |
| Jiang et al.,2017^36^ | 57 (20) | 80 | 21.0 | 22.4 | 14.8 | 21.1 | 35 | 51 Depressed, 6 Euthymic |  | 3.0 T | DPARSFA | 6 | *P* < 0.05 (corrected) | 10 |
| Li et al., 2017a^37^ | 20 (13) | 20 (13) | 31.0 | 30.0 | NA | NA | NA | Depressed |  | 3.0 T | DPARSF | 4 | *p* < 0.05（Alphasim） | 8.5 |
| Li et al., 2017b^38^ | 25 (14) | 24 (14) | 29.5 | 25.8 | 63.6 | 20.9 | 68 | Depressed |  | 3.0 T | DPARSF | NA | *p* < 0.05（Alphasim） | 9.5 |
| Geng et al., 2017^#39^ | 19 (11) | 20 (10) | 33.4 | 32.1 | NA | NA | NA | Depressed |  | 3.0 T | DPARSF | 4 | *p* < 0.05（Alphasim） | 8.5 |
|  | 20 (7) |  | 32.4 |  | NA | NA | NA | Depressed |  |  |  |  |  |  |
| Song et al., 2017^40^ | 10 (3) | 10 (5) | 47.2 | 42.3 | NA | NA | 0 | Depressed |  | 3.0 T | SPM8 | 4 | *p* < 0.005 (uncorrected) | 9 |
| Steffens et al., 2017^41^ | 52 (14) | 36 (9) | 71.4 | 74.3 | NA | NA | NA | Depressed |  | 3.0 T | DPABI | 6 | *p* < 0.05 (FDR) | 9 |
| Zuo et al., 2017^42^ | 48 (24) | 23 (11) | 16.0 | 17.0 | NA | NA | 0 | Depressed |  | 3.0 T | REST | NA | *p* < 0.05（Alphasim） | 9 |
| Li et al., 2018a^43^ | 27 (14) | 27 (15) | 33.8 | 32.2 | 10.0 | 20.6 | 0 | Depressed |  | 3.0 T | REST | 6 | *p* < 0.05 (GRF) | 10 |
| Li et al., 2018b^44^ | 20 (13) | 20 (13) | 30.7 | 30.5 | NA | 28.3 | 100 | Depressed |  | 3.0 T | DPARSF | 4 | *p* < 0.05（Alphasim） | 9.5 |
| Pan et al., 2018^45^ | 24 (13) | 26 (13) | 35.3 | 34.4 | NA | 24~42 | 0 | Depressed |  | 3.0 T | REST | 4 | *p* < 0.05（Alphasim） | 9 |
| Teng et al., 2018^46^ | 25 (25) | 13 (13) | 35.8 | 38.2 | 6.3 | 25.7 | 0 | Depressed |  | 3.0 T | REST | 4 | *p* < 0.05（Alphasim） | 10 |
| Yang et al., 2018^#47^ | 13 (7) | 14 (7) | 31.2 | 31.8 | NA | 3.9 | 0 | Depressed |  | 3.0 T | REST | 4 | *p* < 0.001 uncorrected | 9.5 |
|  | 13 (7) |  | 31.2 |  | NA | 3.9 | 0 | Remitted |  |  |  |  |  |  |
| Yao et al., 2018^48^ | 31 (10) | 63 | 24.4 | 26.0 | 21.8 | 15.6 | 71 | Depressed |  | 3.0 T | DPABI | 6 | *P* < 0.05 (Alphasim) | 10 |
| Cheng et al., 2019^49^ | 72 (33) | 78 (35) | 22.4 | 22.2 | 0.9 | 22.3 | 0 | Depressed |  | 3.0 T | REST | 8 | *p* < 0.08 (FDR) | 10 |
| Li et al., 2019^#50^ | 28 (7) | 30 (12) | 32.5 | 35.7 | 16.6 | 26.0 | NA | Depressed |  | 3.0 T | Dynamic BC | 8 | *p* < 0.05（FWE） | 9.5 |
|  | 20 (4) |  | 37.1 |  | 19.2 | 22.2 | NA | Depressed |  |  |  |  |  |  |
| **Samples from BD patients** | | | | | | | | | | | | | |  |
|  | **BD** | **HC** | **BD** | **HC** |  |  |  |  |  |  |  |  |  |  |
|  | Subjects (males), n |  | Mean age, years |  | Illness duration, months | HAMD-17 | YMRS | State | Subtype | Scanner | Software | FWHM, mm | Threshold |  |
| Liu et al., 2012^51^ | 26 (9) | 26 (10) | 32.4 | 31.9 | 50.4 | 19.7 | NA | Depressed | NA | 3.0 T | REST | 4 | *P*＜0.01 uncorrected | 9.5 |
| Jiang et al., 2014^15^ | 8 (5) | 25 (12) | 32.5 | 30.5 | NA | 21.0 | NA | Depressed | NA | 3.0 T | DPARSF | 6 | *P* < 0.05 uncorrected | 9 |
| Liu et al.,2014^52^ | 36 (13) | 29 (13) | 36.9 | 38.1 | NA | 9.7 | 22.6 | 19 manic, 17 euthymic | BD I 25, BD Ⅱ 11 | 3.0 T | NA | 6 | *p* < 0.05 uncorrected | 9 |
| Xu et al., 2014^53^ | 29 (18) | 29 (13) | 30.5 | 31.4 | NA | 9.7 | 6.5 | 5 Depressed, 6 manic/mixed or hypomanic, 18 euthymic | NA | 3.0 T | REST | 6 | *p* < 0.05 Alphasim | 9.5 |
| Zhou et al.,2014^54^ | 40 (24) | 40 (23) | 28.1 | 28.3 | 61.6 | 9.9 | 9.9 | NA | NA | 3.0 T | REST | 6 | *p* < 0.05 Alphasim | 9 |
| Lui et al.,2015^55^ | 57 (18) | 59 (26) | 34.0 | 38.0 | 16.9 | NA | 5.6 | NA | NA | 3.0 T | REST | 8 | *p* < 0.05 Alphasim | 8.5 |
| Zou et al.,2015^56^ | 30 (17) | 30 (17) | 23.3 | 23.6 | 12.2 | 6.0 | NA | 23 Manic, 3 Depressed, 2 Mixed, 2 Euthymic | BD I | 3.0 T | REST | 6 | *p* < 0.05 Alphasim | 8 |
| Cui et al., 2016^57^ | 40 (23) | 40 (24) | 28.0 | 28.4 | NA | 9.8 | 9.8 | NA | NA | 3.0 T | DPARSFA | 6 | *p* < 0.05 (Corrected) | 9 |
| Wei et al., 2016^30^ | 12 | 11 | 25.8 | 29.7 | NA | 22.8 | NA | NA | NA | 3.0 T | DPARSF | 4 | *P* < 0.05 (Alphasim) | 8.5 |
| He et al., 2017^35^ | 22 (14) | 22 (13) | 37.6 | 35.1 | NA | 27.0 | NA | Depressed | NA | 3.0 T | DPARSF | 4 | *P* < 0.01 (Alphasim) | 8.5 |
| Jiang et al.,2017^36^ | 46 (19) | 80 (29) | 22.3 | 22.4 | 27.9 | 10.8 | 10.1 | 10 Manic, 1 Hypomanic, 1 Mixed, 15 Depressed, 19 Euthymic | 40 BD I, 6 BD II | 3.0 T | DPARSFA | 6 | *P* < 0.05 (corrected) | 10 |
| Fei et al., 2018^58^ | 17 (17) | 18 | 17~37^*^ | 17~37^*^ | NA | NA | NA | Euthymic | NA | 3.0 T | REST | 6 | *p* < 0.05 GRF | 9.5 |
| Yao et al., 2018^48^ | 20 (5) | 63 (23) | 25.5 | 26.0 | 40.4 | 15.6 | NA | Depressed | NA | 3.0 T | DPABI | 6 | *P* < 0.05 (Alphasim) | 10 |
| Zhang et al., 2018^59^ | 21 (7) | 21 (7) | 25.8 | 25.5 | NA | 24.0 | NA | Depressed | BD II | 3.0 T | DPARSF | 4 | *p* < 0.05 GRF | 9 |
| Zhong et al., 2019^60^ | 90 (48) | 100 (45) | 26.7 | 28.3 | 48.0 | 27.1 | 2.7 | Depressed | BD II | 3.0 T | DPABI | 4 | p < 0.05 GRF | 10 |

Note: Unless otherwise indicated, data are means.

^#^ two datasets included; * data are range; ^Quality score out of 10.

Abbreviations: MDD = major depressive disorder; BD = bipolar disorder; HC = healthy control; NA = not available; NOS = not otherwise specified; FWHM = full width at half maximum; SPM = statistical parametric mapping; REST = the resting-state fMRI data analysis toolkit; FWE = family wise error; FDR = false discovery rate; DPARSF = data processing assistant for resting-State fMRI software; GRF = Gaussian random field; DPABI = data processing & analysis of brain imaging; HAMD = Hamilton depression rating scale; YMRS = Young mania rating scale score.

We conducted subgroup analysis of ALFF difference **a)** between unmedicated MDD and HCs (including 33 studies), **b)** unmedicated BD and HCs (including 3 studies), and regarding ALFF difference **a)** between depressed MDD and HCs (including 46 studies), **b)** depressed BD and HCs (including 6 studies. The results are shown in figure S1, figure S2, and Table S3, Table S4.

**Figure S1** Meta-analyses results regarding ALFF difference **a)** between unmedicated MDD and HCs, **b)** unmedicated BD and HCs. Areas with decreased ALFF value are displayed in blue, and areas with increased ALFF value are displayed in red. The color bar indicates the maximum and minimum SDM-Z values. Abbreviations: HCs, healthy controls; MDD, major depressive disorder; BD, bipolar disorder; SDM, seed-based *d* mapping.


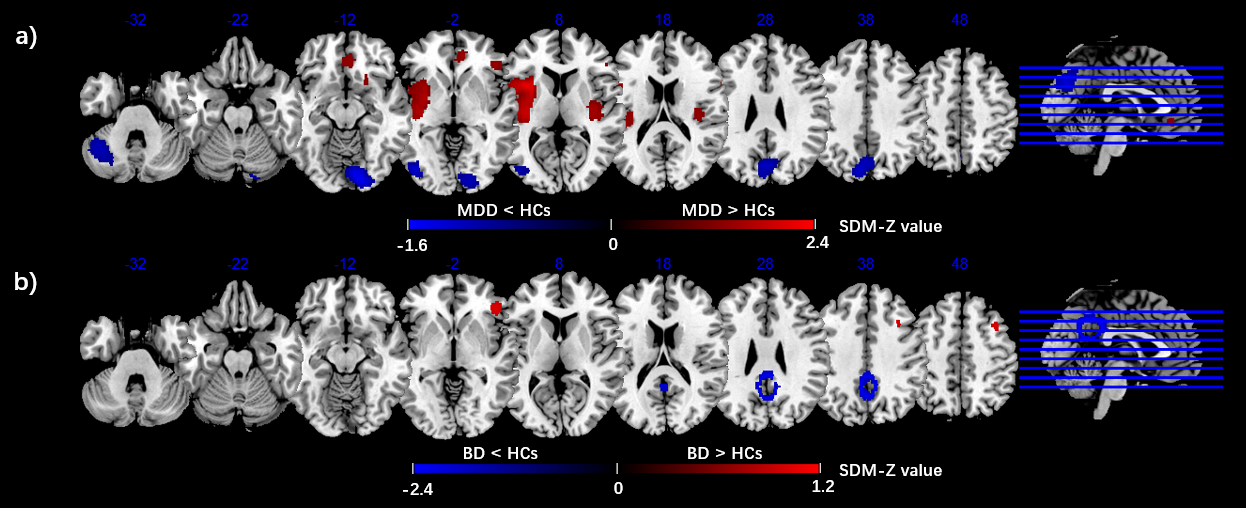


**Figure S2** Meta-analyses results regarding ALFF difference **a)** between depressed MDD and HCs, **b)** depressed BD and HCs. Areas with decreased ALFF value are displayed in blue, and areas with increased ALFF value are displayed in red. The color bar indicates the maximum and minimum SDM-Z values. Abbreviations: HCs, healthy controls; MDD, major depressive disorder; BD, bipolar disorder; SDM, seed-based *d* mapping.


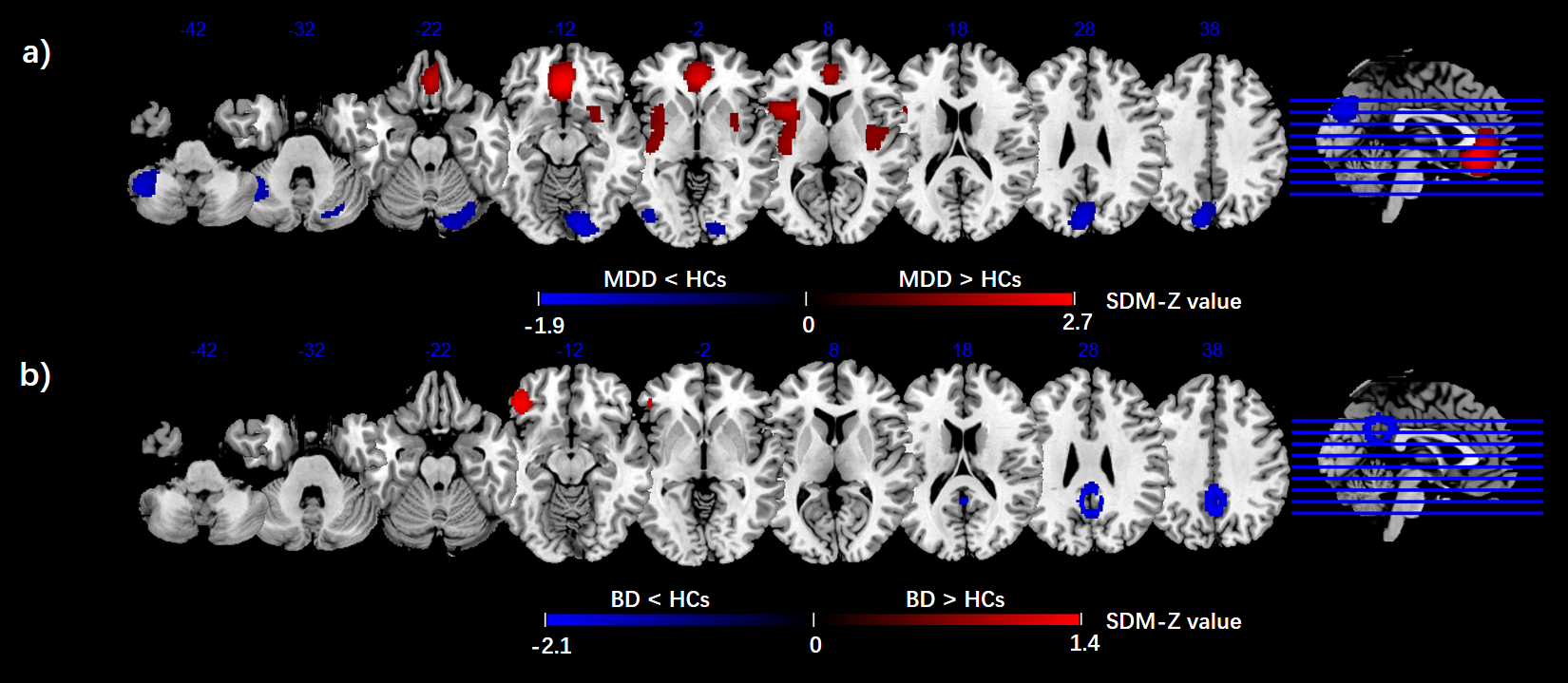


**Table S3** Meta-analyses results regarding ALFF difference between unmedicated MDD and HCs, and between unmedicated BD and HCs, respectively.

| **Local Maximum** |  |  |  | **Cluster** |  |
| --- | --- | --- | --- | --- | --- |
| **Region** | **Peak MNI coordinate**  **(x, y, z)** | **SDM-Z value** | ***p* value** | **No. of voxels** | **Breakdown (No. of voxels)** |
| ***MDD vs. HCs (MDD > HCs)*** |  |  |  |  |  |
| Left insula, BA 48 | -36, 12, 12 | 2.38 | <e-10 | 2742 | Left rolandic operculum, BA 48 (246)  Left lenticular nucleus, putamen, BA 48 (250)  Left superior temporal gyrus, BAs 41 48 (205)  Left heschl gyrus, BA 48 (128)  Left inferior frontal gyrus, opercular part, BA 48 (120)  Left precentral gyrus, BA 6, 44 (118)  Left superior longitudinal fasciculus III (99)  Left supramarginal gyrus, BA 48 (34)  Left inferior frontal gyrus, triangular part, BA 48 (20) |
| Right insula, BA 48 | 36, -18, 8 | 1.58 | 4.80e-4 | 462 | Right heschl gyrus, BA 48 (74)  Right rolandic operculum, BA 48 (41)  Corpus callosum (36)  Right lenticular nucleus, putamen, BA 48 (22) |
| Right superior frontal gyrus, medial orbital, BA 11 | 8, 40, -6 | 1.48 | 9.96e-4 | 330 | Right anterior cingulate / paracingulate gyri (92)  Right gyrus rectus, BA 11 (22) |
| Right supplementary motor area, BA 6 | 8, -8, 68 | 1.58 | 4.85e-4 | 201 |  |
| Right gyrus rectus, BA 11 | 20, 14, -16 | 1.36 | 2.07e-3 | 130 |  |
| Right inferior frontal gyrus, triangular part, BA 47 | 44, 30, -2 | 1.40 | 1.63e-3 | 117 | Right inferior frontal gyrus, orbital part, BA 47 (31) |
| ***MDD vs. HCs (MDD < HCs)*** |  |  |  |  |  |
| Right cerebellum, hemispheric lobule VI, BA 18 | 16, -86, -16 | -2.00 | <e-10 | 1097 | Right lingual gyrus, BAs 17 18 (402)  Right calcarine fissure / surrounding cortex, Bas 17 18 (149)  Right cerebellum, crus I, BA 18 (59)  Right inferior occipital gyrus, BA 18 (58)  Left calcarine fissure / surrounding cortex, BA 17 (22) |
| Left cuneus cortex | -2, -76, 32 | -1.66 | 1.34e-4 | 893 | Left precuneus, BA 7 (212)  Right precuneus, BA 7 (127)  Right cuneus cortex (105)  Right superior occipital gyrus, BA 19 (21) |
| Left cerebellum, crus I | -40, -54, -34 | -1.44 | 5.57e-4 | 900 | Left cerebellum, crus II (98)  Left cerebellum, hemispheric lobule VI (112)  Left cerebellum, hemispheric lobule VIIB (32) |
| Left middle occipital gyrus, BA 19 | -36, -78, 2 | -1.70 | 1.08e-4 | 391 | Left inferior occipital gyrus, BA 19 (83) |
| ***BD vs. HCs (BD > HCs)*** |  |  |  |  |  |
| Right inferior frontal gyrus, triangular part, BA 47 | 44, 32, -2 | 1.0327 | 1.28e-3 | 122 | Right inferior frontal gyrus, orbital part, BA 47 (29) |
| Right superior frontal gyrus, dorsolateral, BA 8 | 26, 12, 60 | 1.12 | 7.28e-4 | 101 |  |
| Right middle frontal gyrus, BA 9 | 38, 14, 50 | 1.05 | 1.12e-3 | 50 |  |
| ***BD vs. HCs (BD < HCs)*** |  |  |  |  |  |
| Left precuneus, BA 23 | -6, -52, 34 | -2.37 | 5.19e-6 | 1080 | Right precuneus, BA 23 (63)  Left posterior cingulate gyrus, BA 23 (93)  Right median cingulate / paracingulate gyri (118)  Left median cingulate / paracingulate gyri (88)  Right posterior cingulate gyrus, BAs 23 26 (33) |

**Abbreviations:** HCs, healthy controls; MDD, major depressive disorder; BD, bipolar disorder; MNI, Montreal Neurological Institute; SDM, seed-based *d* mapping; BA, Brodmann area.

**Table S4** Meta-analyses results regarding ALFF difference between depressed MDD and HCs, as well as between depressed BD and HCs, respectively.

| **Local Maximum** |  |  |  | **Cluster** |  |
| --- | --- | --- | --- | --- | --- |
| **Region** | **Peak MNI coordinate**  **(x, y, z)** | **SDM-Z value** | ***p* value** | **No. of voxels** | **Breakdown (No. of voxels)** |
| ***MDD vs. HCs (MDD > HCs)*** |  |  |  |  |  |
| Right superior frontal gyrus, medial orbital, BA 11 | 4, 36, -12 | 2.63 | <e-10 | 2196 | Left gyrus rectus, BA 11 (286)  Right gyrus rectus, BA 11 (216)  Left superior frontal gyrus, medial orbital, BAs 10, 11 (256)  Left anterior cingulate / paracingulate gyri, BAs 10, 11, 24, 32 (418)  Right anterior cingulate / paracingulate gyri, BAs 10, 11, 24, 32 (246) |
| Left superior longitudinal fasciculus III | -36, 10, 12 | 2.41 | 5.19e-6 | 1965 | Left insula, BA 48 (622)  Left lenticular nucleus, putamen, BA 48 (215)  Left suparmarginal gyrus, BA 48 (131)  Left heschl gyrus, BA 48 (131)  Left superior temporal gyrus, BAs 42, 48 (152)  Left inferior frontal gyrus, opercular part, BA 48 (76)  Left striatum (33) |
| Right olfactory cortex, BA 48 | 28, 8, -14 | 1.53 | 6.14e-4 | 818 | Right insula, BA 48 (273)  Right lenticular nucleus, puatamen, BA 48 (195)  Right heschl gyurs, BA 48 (40) |
| Left precentral gyrus, BA 6 | -48, 2, 42 | 1.30 | 2.52e-3 | 23 |  |
| ***MDD vs. HCs (MDD < HCs)*** |  |  |  |  |  |
| Right cerebellum, hemispheric lobule VI, BA 18 | 16, -84, -16 | -1.82 | 6.19e-5 | 1395 | Right lingual gyrus, BA 18 (366)  Right cerebellum, crus I (351)  Right fusiform, BAs 18, 19 (125)  Right calcarine fissure / surrounding cortex, BAs 17, 18 (63)  Right inferior occipital gyrus, BAs 18, 19 (58)  Right cerebellum, crus II (24) |
| Left cerebellum, crus I | -42, -54, -34 | -1.60 | 1.50e-4 | 991 | Left cerebellum, crus II (129)  Left cerebellum, hemispheric lobule VIIB (70)  Left cerebellum, hemispheric lobule VIII (62)  Left cerebellum, hemispheric lobule VI, BA (78) |
| Left cuneus cortex, BA 18 | -6, -84, 32 | -1.76 | 8.26e-5 | 902 | Left precuneus, BA 7 (98)  Right cuneus cortex (76)  Left superior occipital gyrus, BA 19 (32)  Right precuneus, BA 7 (18) |
| Left inferior network, inferior longitudinal fasciculus | -36, -78, 2 | -1.39 | 6.09e-4 | 169 | Left middle occipital gyrus, BA 19 (96)  Left inferior occipital gyrus, BA 19 (26) |
| *Right precuneus, BA 5* | 6, -58, 58 | -1.16 | 2.42e-4 | 34 |  |
| ***BD vs. HCs (BD > HCs)*** |  |  |  |  |  |
| Left inferior network, inferior fronto-occipital fasciculus | -44, 34, -10 | 1.40 | 4.33e-4 | 450 | Left inferior frontal gyrus, orbital part, BA 47 (311)  Left inferior frontal gyrus, orbital part (24) |
| Right precentral gyrus, BA 4 | 38, -16, 58 | 1.14 | 2.58e-3 | 170 |  |
| ***BD vs. HCs (BD < HCs)*** |  |  |  |  |  |
| Right posterior cingulate gyrus, BA 26 | 4, -42, 26 | -2.04 | 3.61e-4 | 1049 | Left precuneus (260)  Right precuneus (252)  Left posterior cingulate gyrus (128)  Right median cingulate / paracingulate gyri (117)  Left median cingulate / paracingulate gyri (117) |
| Left rolandic operculum, BA 48 | -46, -18, 12 | -1.45 | 4.29e-3 | 22 |  |

**Abbreviations:** HCs, healthy controls; MDD, major depressive disorder; BD, bipolar disorder; MNI, Montreal Neurological Institute; SDM, seed-based *d* mapping; BA, Brodmann area.

**References:**

1. Bai LJ. *Association between glycogen synthese kinase 3-beta gene and major depressive disorder resting state functional magnetic resonance imaging (in Chinese)*: Shanxi Medical University, 2010.

2. Ding J. *Magnetic resonance imaging case control study on brain three-dimension structural abnormalities of first-episode medication-naïve adolescents with major depressive disorder (in Chinese)*: Central South University, 2010.

3. Xu C and Yan BY**.** fMRI study on the spontaneous activity of the brain in primary depression patients and their immediate family members (in Chinese). *Journal of Practical Medical Imaging*. **11**, 69-72 (2010).

4. Jiao Q, et al. Increased activity imbalance in fronto-subcortical circuits in adolescents with major depression. *Plos One*. **6**, e25159 (2011).

5. Wang L, et al. Amplitude of low-frequency oscillations in first-episode, treatment-naive patients with major depressive disorder: a resting-state functional MRI study. *Plos One*. **7**, e48658 (2012).

6. Zhu Z, et al. Spatial patterns of intrinsic neural activity in depressed patients with vascular risk factors as revealed by the amplitude of low-frequency fluctuation. *Brain Res*. **1483**, 82-88 (2012).

7. Fan T, Wu X, Yao L, and Dong J**.** Abnormal baseline brain activity in suicidal and non-suicidal patients with major depressive disorder. *Neurosci Lett*. **534**, 35-40 (2013).

8. Guo LL. *Abnormalities during the resting-state functional magnetic resonance imaging in treatment-naive patients with major depression disorder (in Chinese)*: Shanxi Medical University, 2013.

9. Guo WB, et al. Reversal alterations of amplitude of low-frequency fluctuations in early and late onset, first-episode, drug-naive depression. *Prog Neuropsychopharmacol Biol Psychiatry*. **40**, 153-159 (2013).

10. Jing B, et al. Difference in amplitude of low-frequency fluctuation between currently depressed and remitted females with major depressive disorder. *BRAIN RES*. **1540**, 74-83 (2013).

11. Li XL. *Study brain function on resting-state fMRI and VBM of major depressive disorder (in Chinese)*: Kunming Medical University, 2013.

12. Yang HC. *The clinical and resting-state brain fMRI study about the hypomanic symptoms of the patients with mood disorders (in Chinese)*: Central South University, 2013.

13. Gong Y, et al. Case-control resting-state fMRI study of brain functioning among adolescents with first-episode major depressive disorder. *Shanghai Arch Psychiatry*. **26**, 207-215 (2014).

14. Jia AX, et al. Research of Brain Functional Magnetic Resonance Imaging on Patients with First-episode Major Depressive Disorder (in Chinese). *Medical Innovation of China*., 1-3, 4 (2014).

15. Jiang XW. *Comparative Study of Amplitude of Low-frequency Fluctuations in Prefrontal Cortex and Cingulate Cortex of First-episode Bipolar Disorder Depression and First-episode Major Depressive Disorder：A Resting-State Functional Magnetic Resonance Imaging Study (in Chinese)*: China Medical University, 2014.

16. Liu J, et al. Alterations in amplitude of low frequency fluctuation in treatment-naive major depressive disorder measured with resting-state fMRI. *Hum Brain Mapp*. **35**, 4979-4988 (2014).

17. Qiu HT, et al. Research on Amplitude of Low-frequency Fluctuation in Patients with Major Depression Based on Resting-state Functional Magnetic Resonance Imaging (in Chinese). *Journal of Biomedical Engineering*. **31**, 97-102 (2014).

18. Yan R, Yao ZJ, Wei MB, Tang H, and Lu Q**.** Amplitude of low frequency fluctuation in female depression patients: a resting-state functional magnetic resonance imaging study (in Chinese). *Chinese Journal of Psychiatry*. **47**, 195-199 (2014).

19. Yan R, et al. Amplitude of low frequency fluctuation in first-episode depression and its relationship with the separate symptom clusters: a resting-state fMRI study (in Chinese). *Chinese Journal of Behavioral Medicine and Brain Science*. **23**, 673-675 (2014).

20. Zhang X, et al. First-episode medication-naive major depressive disorder is associated with altered resting brain function in the affective network. *Plos One*. **9**, e85241 (2014).

21. Zhao B, Wei GQ, Hou LY, Xu C, and Zhang KR**.** Resting state functional magnetic resonance imaging in patients with first-episode major depression disorder (in Chinese). *Chinese Journal of Behavioral Medicine and Brain Science*. **23**, 334-337 (2014).

22. Fang J, et al. Functional and Anatomical Brain Abnormalities and Effects of Antidepressant in Major Depressive Disorder: Combined Application of Voxel-Based Morphometry and Amplitude of Frequency Fluctuation in Resting State. *J Comput Assist Tomogr*. **39**, 766-773 (2015).

23. Li LS, et al. A association study of norepinephrine transporter (NET) gene polymorphism and A resting-state fMRI in major depressive disorder (in Chinese). *Chinese Journal of Clinicians (Electronic Edition)*., 1534-1539 (2015).

24. Qiu TS, Dai RJ and Liu YJ**.** Amplitude of Low-Frequency Fluctuation in First-Episode Depressed Patients on Resting-State Functional Magnetic Resonance Imaging (in Chinese). *Journal of Data Acquisition & Processing*. **30**, 940-947 (2015).

25. Yong N, et al. Resting-state fMRI study on alteration of amplitude of low frequency fluctuation and its relationship with aggressive behaviors in first-episode major depressive disorder patients (in Chinese). *Academic Journal of Second Military Medical University*. **36**, 261-267 (2015).

26. Du L, et al. Early life stress affects limited regional brain activity in depression. *Sci Rep*. **6**, 25338 (2016).

27. Guo DL, Gao Y, Niu GM, and Xie SH**.** First-episode depression resting state of brain function low frequency amplitude research (in Chinese). *Chin J Magn Reson Imaging*. **7**, 407-411 (2016).

28. Wang L, et al. Frequency-dependent changes in amplitude of low-frequency oscillations in depression: A resting-state fMRI study. *Neurosci Lett*. **614**, 105-111 (2016).

29. ***Wang XL.*** *Resting-state functional magnetic resonance imaging of neural activity in hypothalamus and prefrontal lobe - limbic network in depressed patients (in Chinese)*: Hangzhou Normal University, 2016.

30. Wei QX. *The differences of male unipolar depressiong and bipolar depressiong: a resting_state fMRI study (in Chinese)*: Nanjing University, 2016.

31. Zhang X, et al. Imbalanced spontaneous brain activity in orbitofrontal-insular circuits in individuals with cognitive vulnerability to depression. *J Affect Disord*. **198**, 56-63 (2016).

32. Zhang X, et al. Altered neuronal spontaneous activity correlates with glutamate concentration in medial prefrontal cortex of major depressed females: An fMRI-MRS study. *J Affect Disord*. **201**, 153-161 (2016).

33. Zhao B, et al. Study regional homogeneity and low frequency amplitude of brain resting state functional magnetic resonance imaging in patients with depression (in Chinese). *Chinese Journal of Behavioral Medicine and Brain Science*. **25**, 986-991 (2016).

34. Zhu XL, Chen L and Yuan FL**.** Amplitude of Low Frequency-fluctuation in Young MDD Patients: A Resting-state fMRI Study (in Chinese). *Chinese Journal of Clinical Psychology*. **24**, 805-807 (2016).

35. He XT. *A resting-state fMRI study of bipolar disorder with onset of major depressive episode (in Chinese)*: Shanxi Medical University, 2017.

36. Jiang X, et al. Alteration of cortico-limbic-striatal neural system in major depressive disorder and bipolar disorder. *J Affect Disord*. **221**, 297-303 (2017).

37. Li P, Li F, Wang CM, Bo QJ, and Wang CY**.** The characteristic of amplitude of low frequency fluctuation and fractional amplitude of low frequency fluctuation in early-onset depression:a resting-state functional MRI study (in Chinese). *Shanxi Medical Journal*. **46**, 2158-2162 (2017).

38. Li YH, Liu XZ, Liu YJ, Liu ZN, and Tang H**.** The Functional Magnetic Resonance Imaging of Major Depressive Disorder in Resting State and Task State (in Chinese). *Chinese Journal of Clinical Psychology*. **25**, 393-399, 405 (2017).

39. Geng JT, et al. Amplitude of low frequency fluctuation in depressive patients with diurnal rhythm: a resting-state fMRI study (in Chinese). *Chinese Journal of Behavioral Medicine and Brain Science*. **26**, 605-609 (2017).

40. Song YL, Sun SG, Song XL, Mao N, and Wang B**.** BOLD-fMRI study on the basic activity of the brain in major disorder depression and their first-degree relatives (in Chinese). *Journal of Practical Radiology*. **33**, 653-657 (2017).

41. Steffens DC, Wang L, Manning KJ, and Pearlson GD**.** Negative Affectivity, Aging, and Depression: Results From the Neurobiology of Late-Life Depression (NBOLD) Study. *Am J Geriatr Psychiatry*. **25**, 1135-1149 (2017).

42. Zuo WB. *Relationship between 5-HTTLPR Gene Polymorphism and Brain Function and Cognitive Function in Adolescents with First-episode Major Depressive Disorder (in Chinese)*: Zhengzhou University, 2017.

43. Li G, Rossbach K, Zhang A, Liu P, and Zhang K**.** Resting-state functional changes in the precuneus within first-episode drug-naive patients with MDD. *Neuropsychiatr Dis Treat*. **14**, 1991-1998 (2018).

44. Li P, et al. A relevant research between spontaneous activity of resting state of brain area and severity of illness of patients with EOD under functional magnetic resonance imaging (in Chinese). *China Medical Equipment*. **15**, 61-66 (2018).

45. Pan MJ, et al. Study on resting-state fMRI based on amplitude of low-frequency fluctuation in patients with major depression (in Chinese). *Chinese Journal of Contemporary Neurology and Neurosurgery*. **18**, 171-176 (2018).

46. Teng C, et al. Abnormal resting state activity of left middle occipital gyrus and its functional connectivity in female patients with major depressive disorder. *BMC Psychiatry*. **18**, 370 (2018).

47. Yang C, et al. Identify abnormalities in resting-state brain function between first-episode, drug-naive major depressive disorder and remitted individuals: a 3-year retrospective study. *Neuroreport*. **29**, 907-916 (2018).

48. Yao XD. *Study of Bipolar Disorder and Major Depressive Disorder during Depressive State on Resting-state Functional Magnetic Resonance Imaging and Correlation with Brain-Derived Neurotrophic Factor (in Chinese)*: China Medical University, 2018.

49. Cheng C, et al. State-Related Alterations of Spontaneous Neural Activity in Current and Remitted Depression Revealed by Resting-State fMRI. *Front Psychol*. **10**, 245 (2019).

50. Li J, Duan X, Cui Q, Chen H, and Liao W**.** More than just statics: temporal dynamics of intrinsic brain activity predicts the suicidal ideation in depressed patients. *Psychol Med*. **49**, 852-860 (2019).

51. Liu CH, et al. Abnormal baseline brain activity in bipolar depression: a resting state functional magnetic resonance imaging study. *Psychiatry Res*. **203**, 175-179 (2012).

52. Liu H. *Multi-modal MRI study of brain in subj ects with mental and neurological dlsorders (in Chinese)*: China Medical University, 2014.

53. Xu K, et al. Amplitude of low-frequency fluctuations in bipolar disorder: a resting state fMRI study. *J Affect Disord*. **152-154**, 237-242 (2014).

54. Zhou Q. *Comparison of Amplitude of Low Frequency Fluctuation between Schizophrenia and Bipolar Disorder Patients：A Resting--state Functional Magnetic Resonance Imaging Study (in Chinese)*: China Medical University, 2014.

55. Lui S, et al. Resting-state brain function in schizophrenia and psychotic bipolar probands and their first-degree relatives. *Psychol Med*. **45**, 97-108 (2015).

56. Zou WJ, et al. Resting state amplitude of low frequency fluctuation study in young patients with early stage bipolar disorder Ⅰ (in Chinese). *Chinese Journal of Medical Imaging Technology*. **31**, 976-980 (2015).

57. Cui WH, Zhou Q, Wang F, and Tang YQ**.** Comparison of Amplitude of Low Frequency Fluctuation between Schizophrenia and Bipolar Disorder Patients: a Resting-state Functional Magnetic Resonance Imaging Study (in Chinese). *Journal of China Medical University*. **45**, 977-981, 984 (2016).

58. Fei LC, Wu Q and Wang Y**.** Amplitude of low frequency fluctuation and functional connectivity on patients with euthymic bipolar disorder:a resting-state fMRI study (in Chinese). *J Clin Psychiat*. **28**, 321-325 (2018).

59. Zhang P, et al. Amplitude of low-frequency fluctuation and functional connectivity analysis-based resting-state functional magnetic resonance imaging study in bipolar Ⅱ depression patients (in Chinese). *Chinese Journal of Psychiatry*. **51**, 105-112 (2018).

60. Zhong S, et al. Correlation between Intrinsic Brain Activity and Thyroid-Stimulating Hormone Level in Unmedicated Bipolar II Depression. *Neuroendocrinology*. **108**, 232-243 (2019).
